# Large cell non-Hodgkin's lymphoma masquerading as renal carcinoma with inferior vena cava thrombosis: a case report

**DOI:** 10.1186/1752-1947-5-245

**Published:** 2011-06-28

**Authors:** Erika E Samlowski, Christopher Dechet, Alan Weissman, Wolfram E Samlowski

**Affiliations:** 1 Nevada Cancer Institute, One Breakthrough Way, Las Vegas, NV 89135, USA; 2 Huntsman Cancer Institute, 2000 Circle of Hope Drive, Salt Lake City, UT 84148, USA; 3 2435 Grassy Spring Pl, Las Vegas NV 89135, USA

## Abstract

**Introduction:**

Many cancers are associated with inferior vena cava (IVC) obstruction, but very few cancers have the ability to propagate within the lumen of the renal vein or the IVC. Renal cell carcinoma is the most common of these cancers. Renal cancer with IVC extension has a high rate of recurrence and a low five year survival rate.

**Case presentation:**

A 62-year-old Caucasian woman previously in good health developed the sudden onset of severe reflux symptoms and right-sided abdominal pain that radiated around the right flank. A subsequent ultrasound and CT scan revealed a right upper pole renal mass with invasion of the right adrenal gland, liver, left renal vein and IVC. This appeared to be consistent with stage III renal cancer with IVC extension. Metastatic nodules were believed to be present in the right pericardial region; the superficial anterior abdominal wall; the left perirenal, abdominal and pelvic regions; and the left adrenal gland. The pattern of these metastases, as well as the invasion of the liver by the tumor, was thought to be atypical of renal cancer. A needle biopsy of a superficial abdominal wall mass revealed a surprising finding: The malignant cells were diagnostic of large-cell, B-cell non-Hodgkin's lymphoma. The lymphoma responded dramatically to systemic chemotherapy, which avoided the need for nephrectomy.

**Conclusion:**

Lymphomas only rarely progress via intraluminal vascular extension. We have been able to identify only one other case report of renal lymphoma with renal vein and IVC extension. While renal cancer would have been treated with radical nephrectomy and tumor embolectomy, large-cell B-cell lymphomas are treated primarily with chemotherapy, and nephrectomy would have been detrimental. It is important to remember that, rarely, other types of cancer arise from the kidney which are not derived from the renal tubular epithelium. These may be suspected if an atypical pattern of metastases or unusual invasion of surrounding organs is present. A preoperative or intraoperative biopsy may be helpful in these cases.

## Introduction

A number of malignancies are associated with inferior vena cava (IVC) obstruction, but very few cancers exhibit a potential for tumor thrombus formation and intravascular extension within the lumen of the IVC. Renal cancer is the most common tumor associated with IVC thrombosis [1,2]. More rarely, IVC extension has been described in case reports of patients with adrenal cancer, hepatoma, advanced testicular cancer, Wilms' tumor, colon cancer, gastric cancer, pancreatic cancer, transitional cell carcinoma of the bladder and peripheral neuroectodermal tumor [3,4].

The incidence of renal vein or IVC thrombosis in patients with renal cancer appears to be 4% to 10% [5]. In the newly revised 2009 American Joint Committee on Cancer staging system, tumor extension into the renal vein or the vena cava is classified as stage T3a if there is renal vein extension, as stage T3b if the tumor extends into the subdiaphragmatic IVC and as stage T3c if renal cancer extends above the diaphragm or invades the endothelium of the IVC [6]. The extent of IVC thrombus can also be sub-classified by the Mayo Clinic system into level I (< 2 cm above the renal vein), stage II (infrahepatic thrombus), stage III (intrahepatic IVC involvement below the diaphragm) and stage IV (above the diaphragm or extending into the right atrium) [7]. The extent of tumor thrombus extension appears to correlate closely with surgical outcome. If the tumor has extended above the diaphragm, the chances for complete surgical resection and consequently patient survival are diminished [8]. Renal cancer with IVC extension is generally treated with radical nephrectomy and tumor embolectomy [2]. Renal cancer with renal vein or IVC involvement remains associated with a high local and distal failure rate [6,9]. Al Otabi *et al. *[8] described a 64% recurrence rate in their 50-patient series, with a 50% five year survival rate. This failure rate is far greater than that for stage T1 or T2 renal cancer (five year survival rates of 81% and 74%, respectively) [6]. A substantial fraction of these patients (36% to 40%) present with concomitant lymph node or distant metastatic disease at the time IVC involvement is detected [5]. The extent to which the tumor has physically invaded the renal vein and the vena cava endothelium (including intrahepatic branches) also may be important to the patient's prognosis, although this is currently a subject of debate.

Primary renal non-Hodgkin's lymphoma (NHL) is thought to be rare, perhaps because of the lack of renal lymphatic tissue [10,11]. There have been a few case reports of intravascular extension of lymphomas. Rarely, NHL can present with focal intravascular lymphoma masses. This syndrome, termed intravascular large B-cell lymphoma, is generally characterized by proliferation of lymphoma cells in smaller blood vessels. In rare patients, intravascular large B-cell lymphoma can present as masses in large blood vessels. A single case of intravascular large B-cell lymphoma that presented initially as superior vena cava syndrome has been reported [12]. Rare additional case reports have described patients who presented with superior vena cava thrombosis which later revealed the presence of Burkitt's lymphoma, lymphoblastic NHL [13] and a primary cardiac B-cell lymphoma that presented as superior vena cava syndrome [14].

## Case presentation

**Figure 1:**
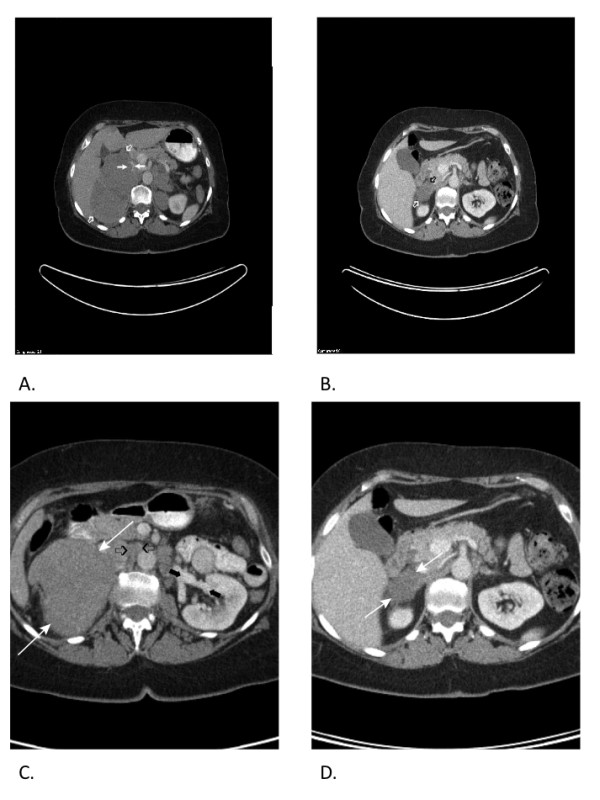
**Right renal mass and inferior vena cava (IVC) thrombus**. Renal mass and IVC thrombus. **(A) **Computed tomographic (CT) image showing large renal mass (open white arrows) invading right adrenal gland, liver and IVC. Note intravascular thrombus in the IVC (solid white arrows). **(B) **CT image showing marked improvement in renal mass after three cycles of cyclophosphamide, doxorubicin, vincristine, prednisone plus rituxumab (R-CHOP) chemotherapy (open arrows). **(C) **CT image showing normal left renal vein (black solid arrow), but the right renal vein is not seen (black open arrows), consistent with occlusion by malignant thrombus. White arrows identify the renal tumor. **(D) **CT image shows improvement of right renal mass.

**Figure 2:**
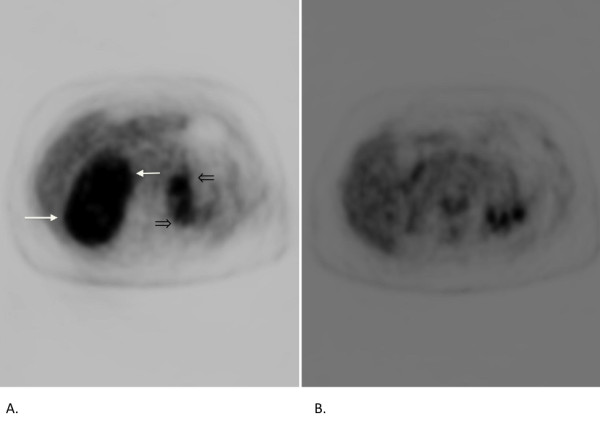
**Positron emission tomographic (PET) scan uptake in renal mass and abdominal metastasis**. Axial PET image. **(A) **Intense fluorodeoxyglucose activity is seen in right renal mass (white arrows). Contralateral abdominal disease is also identified (black open arrows). **(B) **Axial PET image showing absent fluorodeoxyglucose activity in right renal mass after three cycles of chemotherapy.

**Figure 3:**
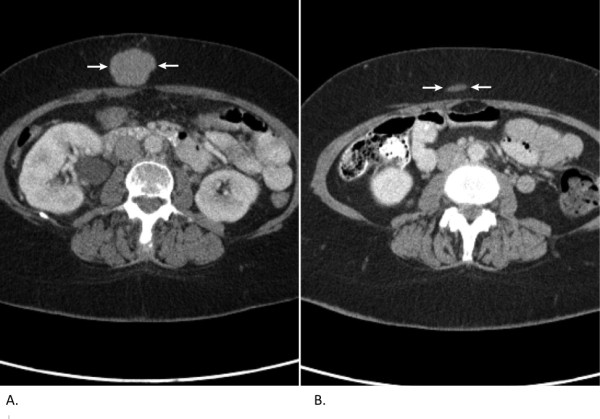
**Anterior abdominal subcutaneous tumor**. Subcutaneous abdominal mass (source of diagnostic biopsy). **(A) **Subcutaneous mass at initial diagnosis (white arrows). **(B) **Marked reduction in subcutaneous mass following three cycles of chemotherapy (white arrows). The CT scan slices are aligned to show the maximum dimensions of the soft tissue tumor mass.

A 62-year-old Caucasian woman who had previously been in good health, except for a history of treated hypothyroidism, presented to our hospital in November 2009 with sudden onset of severe reflux symptoms and right-sided abdominal pain that radiated around the right flank. An abdominal ultrasound examination was performed. This revealed a large right-sided renal mass. A subsequent computed tomographic (CT) scan confirmed a 13 cm × 9 cm right upper pole renal mass with probable invasion of the right adrenal gland and liver. Tumor extension into the left renal vein and the IVC was also observed. This patient's presentation corresponded to Mayo Clinic level III (Figures 1A and 1C). Her clinical presentation appeared to be consistent with a large renal carcinoma with renal vein and IVC extension. Metastatic nodules were believed to be present in the right pericardial region; the anterior abdominal soft tissue left pelvis; the left perirenal, abdominal and pelvic regions; and the left adrenal gland. This pattern of metastasis seemed to be atypical of renal cell carcinoma (RCC). Typical renal metastases are found in the lung, periaortic lymph nodes or bone. This contrasted with the extensive intraabdominal spread seen in our patient. In addition, direct tumor extension into the liver is a rare finding in RCC. This tumor showed strong fluorodeoxyglucose uptake on a subsequent positron emission tomographic (PET) scan (Figure 2A). Upon further questioning, the patient complained of ongoing, mild right flank discomfort, chronic fatigue and rare sweats, but no weight loss or chills. Her physical examination did not reveal a palpable abdominal mass. Baseline complete blood count and blood chemistry testing (including liver and kidney function) were normal, except for an elevated lactate dehydrogenase level of 340IU/L (normal range, 120 to 250IU/L). Because the patient had a superficial abdominal wall mass (Figure 3A), a needle biopsy was performed to aid in surgical treatment planning. The cytology and core biopsy from this specimen revealed a surprising finding: The malignant cells were thought to represent large-cell, B-cell NHL. This was confirmed by flow cytometry, which identified a κ light chain restricted B-cell population that expressed CD19 and CD20. The patient is currently undergoing cyclophosphamide, doxorubicin, vincristine, prednisone plus rituxumab (R-CHOP) chemotherapy. R-CHOP treatment currently represents the most effective chemotherapy regimen for large-cell NHL in patients over 60 years of age [15]. This regimen was well tolerated, and upon reimaging with PET and CT scans after three cycles of chemotherapy, she showed an objective partial response in tumor dimensions (Figures 1B and 1D) with markedly decreased fluorodeoxyglucose uptake (Figure 2B). The anterior abdominal subcutaneous mass also demonstrated a nearly complete response after three cycles of R-CHOP chemotherapy (Figure 3B).

## Discussion

Tumor thrombi in major venous structures can occur as a complication of cancer. Approximately 90% of all cases of superior vena cava syndrome are caused by compression of the superior vena cava by an extrinsic tumor. Most commonly, this is a complication of lung cancer (especially small-cell lung cancer), but it can also be caused by lymphomas, as well as by breast, esophageal, thyroid, thymus and testicular cancer. A very small percentage of cases of superior vena cava syndrome are associated with actual intravascular tumor invasion and extension. Obstruction of the IVC is even less common [3,4]. Extrinsic compression of the IVC by tumors such as testicular cancer, lymphoma, pancreatic cancer, Wilms' tumor and sarcoma may occur, but these are relatively rare events associated with advanced cancer. Intravascular extension and tumor thrombi are most commonly seen in patients with RCC, occurring in 4% to 10% of RCC cases [5]. IVC tumor thrombi can also be caused by adrenal cortical carcinomas and hepatomas and much less frequently by other tumor types [3,4].

The current case is quite unusual. The large renal mass seen on the radiographs of the right kidney was thought to represent RCC, with invasion into the right adrenal gland and liver and extension into the renal vein and the IVC on the basis of PET and CT scan abnormalities not verified by an additional biopsy. Direct extension of RCC into the liver is also thought to be uncommon [16]. A pre-treatment biopsy identified the tumor in our patient to be large-cell, B-cell NHL. Lymphomas very rarely progress via intraluminal vascular extension. We have been able to identify only one other case report of renal vein and IVC extension of a renal lymphoma. That case report, published by Wagner *et al. *[17], described another patient with large-cell, B-cell NHL mimicking stage III renal adenocarcinoma with tumor thrombi in the renal vein and IVC.

## Conclusion

The presence of a renal mass with a tumor extending into the renal vein and IVC is most frequently a manifestation of clear-cell RCC. It should be remembered that other non-renal tubular epithelium-derived cancers occasionally arise from the kidney. Our case report represents the second published instance of a renal lymphoma. Intravascular extension of lymphoma is a rare clinical finding. An atypical pattern of intra-abdominal spread of metastases and liver invasion was an important clue that this was potentially not a RCC. When feasible, a pre-treatment (or intraoperative) biopsy may be helpful in planning appropriate management strategies. While the management of RCC with IVC thrombosis may include radical nephrectomy and tumor embolectomy, this is a difficult operation with significant morbidity and mortality. In contrast, large-cell lymphomas are treated primarily with chemotherapy, such as the current R-CHOP regimen combined with the CD20 monoclonal antibody rituximab (R-CHOP). This regimen results in a very high response rate, as seen in our patient, and 50% to 70% durable complete remission rates (with five-year to ten-year follow-up). A nephrectomy would be unhelpful in treating renal lymphoma. In our opinion, features that would increase suspicion for non-RCC would be extensive effacement of adjacent organs by tumor masses and atypical patterns of metastases.

### Patient's perspective

The occurrence of pain hit quickly right after my indulgence at Thanksgiving dinner. I thought I had reflux from over-eating of rich foods. The pain traveled from the abdomen around to the back and up into and under my right shoulder blade. I was uncomfortable in all positions, whether standing, sitting or lying down. A handful of Tums for several days did not do the trick, and I went to see my physician. A battery of tests was ordered to determine what was happening. During the course of the tests, nodules started popping up in my abdomen, close to the surface. My pain had intensified throughout the abdomen and into the back to the point where I could not stand for any longer than a few minutes without doubling over.

After the ultrasound results came back, it was noted that a "suspicious spot" was seen on my kidney, and subsequent blood work and a CT scan suggested that I had renal cancer. I sought the assistance of the Nevada Cancer Institute for the diagnosis of renal cancer. I had a biopsy, which resulted in the discovery that I had non-Hodgkin's lymphoma. Once the chemotherapy treatments started, my pain subsided along with the nodules, during the second treatment. I have had very mild if any side effects during the chemo. I base this on my prior good health, strong will, positive attitude, a great support team and making sure I stay extremely hydrated during the treatments. I have remained active and working and have had as little disruption to my life as possible. Presently I am feeling very good, have had excellent results from the PET scan after the third chemotherapy, and am awaiting the results of my next scan after the sixth treatment.

## Abbreviations

IVC: Inferior Vena Cava; NHL: Non-Hodgkin's Lymphoma; PET: Positron Emission Tomography.

## Consent

Written informed consent was obtained from the patient for publication of this case report and any accompanying images. A copy of the written consent is available for review by the Editor-in-Chief of this journal.

## Competing interests

The authors declare that they have no competing interests.

## Authors' contributions

ES performed the literature search and wrote the case report. AW interpreted the radiographs and provided publication-quality images. CD provided expert input on urologic management of renal vein thrombosis and renal cancer. WES helped write the manuscript and formatted the document according to *JMCR *standards. All authors helped edit the final manuscript and approved its submission.
